# YTHDF1 boosts the lactate accumulation to potentiate cervical cancer cells immune escape

**DOI:** 10.1038/s41419-024-07128-0

**Published:** 2024-11-18

**Authors:** Jing Xiong, Ling He, Xiaoshan Chai, Yongjing Zhang, Shujuan Sun

**Affiliations:** Department of Obstetrics and Gynecology, The Second Xiangya Hospital of Central South University, Changsha, HN China

**Keywords:** Immune cell death, Cancer epigenetics

## Abstract

Lactate is a major metabolic product of tumor cells in microenvironment. Increasing evidence has indicated that lactate accumulation could alter the immune response in human cancers, including cervical cancer. However, the function and significance of N^6^-methyladenosine (m^6^A) reader YTHDF1 in cervical cancer cells’ lactate metabolism and immunotherapy remain obscure. Results illustrated that YTHDF1 predicted unfavorable clinical outcomes of cervical cancer, which was negatively correlated with CD8^+^ T cell infiltration. In the co-culture of tumor cells with CD8^+^ T cells, YTHDF1 overexpression promoted the lactate accumulation and attenuated the cytotoxic CD8^+^ T cell’s killing effect. Correspondingly, YTHDF1 knockdown exerted the opposite effects. Mechanistically, YTHDF1 targeted the m^6^A site on SLC16A1 gene (MCT1) to determine its fate. YTHDF1 upregulated MCT1 expression by enhancing MCT1 stability mediated by m^6^A-modified manner. Collectively, our results revealed an oncogenic role played by YTHDF1 in cervical cancer through m^6^A/MCT1-dependent manner. In conclusion, these findings unveil the immune escape-promoting effect of YTHDF1 in cervical cancer by boosting the lactate accumulation, which might illuminate a novel target for more precise immunotherapy.

## Introduction

Cervical cancer is one of most common malignancies for reproductive tract in females worldwide, with a global high mortality in developing country [1]. For locally advanced subtype of cervical cancer, the 5-year survival rate for different stages is pessimistic, e.g. 60–65% for IIB stage, 25–50% for IIIB stage, and 20–35% for IV stage respectively [2, 3]. Current research into cancer treatment methods is actively exploring how to improve the drug sensitivity of cervical cancer cells while minimizing damage to the tissue surrounding the normal cervix [4]. This is an important area of research aimed at improving the effectiveness of treatment while having fewer side effects.

Immune escape is a phenomenon in which tumor cells escape immune cell killing and is associated with abnormal function of anti-tumor related immune cells, such as dendritic cells and T lymphocytes [5, 6]. Immune escape can inhibit T cell migration and form immunosuppressive tumor microenvironment. In tumor tissues, immunosuppressive factors inhibit the infiltration of CD8^+^ T cells, forming an immunosuppressive tumor microenvironment [7]. PD-1/PD-L1 signaling pathway is considered to be a key immune barrier pathway to inhibit the activation of CD8^+^ T, and the activation of this pathway can inhibit the recognition and killing function of CD8^+^ T on cancer cells, resulting in cancer cells escaping the surveillance and killing of the immune system [8].

N^6^-methyladenosine (m^6^A) is the most common type of RNA modification that occurs with methylation at the N^6^-position of adenosine nucleotides [9, 10]. In the human cancer tumorigenesis, the functions of m^6^A have been profoundly explored [11]. For example, in cervical cancer, the TRIM11 mRNA’s stability is enhanced by IGF2BP1 depending on m^6^A modification, which is mediated by METTL14 and accelerates cervical cancer progression by mediating PHLPP ubiquitination [12]. Moreover, IGF2BP3 stabilizes the GLS and GLUD1 mRNA through m^6^A modification, thereby facilitating glutamate and glutamine metabolism and regulating lactate production in cervical cancer cells [13]. Therefore, the deeply exploration for m^6^A on cervical cancer plays an important role.

Lactate, which is prominently produced by glycolysis, until recently is regarded as a main fuel oxidation of cancer cells and signal transduction [14]. Lactate has a wide range of biological impacts in tumor microenvironment with low nutrition, hypoxia, and low pH [15]. The exchange of lactate through cellular membranes is gated by monocarboxylate transporters, such as MCT1 and MCT4. Given the vital role of MCT1/4 on tumor progression, the specific focus on lactate metabolism and lactate-induced immune escape is very important [16, 17].

In this study, our findings demonstrated the significant function of YTHDF1 in promoting cervical cancer cells’ immune escape. YTHDF1 upregulated in the cervical cancer tissue and cells and correlated to the poor prognosis and CD8^+^ T infiltration. YTHDF1 binds with the MCT1 via the m^6^A-modified site to enhance its mRNA stability, thereby promoting the lactate accumulation, thereby potentiating cervical cancer cells immune escape in MCT1-dependent manner. Collectively, this study reveals that YTHDF1 may serve as a promising anti-tumor candidate for developing an understanding of antitumor immunity through m^6^A regulation.

## Materials and methods

### Clinic tissue specimens

The tissue samples of cervical cancer for the present study were collected from individuals who underwent operative treatment in The Second Xiangya Hospital of Central South University (Table 1). None of the enrolled individuals had received any antitumor therapy before this study. Two independent pathologists performed the pathological diagnostic reports for each specimens. All the collected individuals were informed before treatment and all of them signed a consent form. This study had been approved by Ethics Committee of The Second Xiangya Hospital of Central South University.

**Table 1:** The relationship between YTHDF1 and clinicopathological characteristics of cervical cancer patients.

	Total	YTHDF1^High^ (*n* = 28)	YTHDF1^Low^ (*n* = 28)	*p*
Age				
≥50	35	18	17	0.782
<50	21	10	11	
HPV status				
HPV+	38	20	18	0.775
HPV−	18	8	10	
Tumor size				
<4 cm	21	6	15	0.013
≥4 cm	35	22	13	
FIGO stages				
I/IIA	33	21	12	0.014
IIB/III/IV	23	7	16	
Tumor differentiation				
Well/moderate	30	19	11	0.032
Poor	26	9	17	
Lymph node metastasis				
Positive	24	13	11	0.589
Negative	32	15	17	

### Cancer cells and transfection

The cervical cancer cell lines (C-33A, HeLa, CaSki, SiHa) and control human epidermal cell (HaCaT) were purchased from Chinese Academy of Sciences Cell Bank (Shanghai, China) and American Type Culture Collection (ATCC, Manassas, VA, USA). Cells were cultured in the RPMI 1640 medium (Invitrogen, Carlsbad, CA) added with fetal bovine serum (10% FBS), 100 U/mL penicillin G and 100 μg/mL streptomycin in 5% CO_2_ at 37 °C atmosphere.

### Oligonucleotides, plasmids and transfection

Oligonucleotides targeting YTHDF1 (sh-YTHDF1) and controls (sh-NC) were designed and synthesized by (Shanghai GenePharma CO. Ltd). The full length of YTHDF1 was cloned into pCDH puro lentiviral vector (CD510B-1, System Biosciences). The siRNA target MCT1 weas synthesized by RiBoBio (Guangzhou, China). The oligonucleotides and plasmids were transfected using Lipofectamine^TM^ 2000 (Invitrogen, Thermo Fisher Scientific, USA) according to the manufacturer’s instructions.

### CD8^+^ T cell coculture assay

Human peripheral blood mononuclear cells (hPBMCs) were isolated from the blood of normal volunteers. hPBMCs were purified using the Ficoll kit (Sigma-Aldrich, USA) according to the manufacture’s instruction. The isolated CD8^+^ T cells were activated using Ultra-LEAF Purified anti-mouse CD3/CD28 (BioLegend) and IL-2 (10 ng/mL) in RPMI-1640 medium for three days according to the manufacturer’s protocol. The transfected cancer cells were incubated with activated CD8^+^ T cell for 48 h. For the coculture system assay, the ratio within cancer cells and CD8^+^ T ranged from 1:1 to 1:10. After coculture, the debris were removed by PBS washing for following assay.

### Real-time quantitative PCR (RT-PCR)

The cells were collected and the total RNA was extracted using the RNA prep Pure Cell kit (Invitrogen). Each mRNA was reversely transcribed using a cDNA synthesis kit (TransGen Biotech Co., Ltd). Reverse transcription PCR (RT-PCR) was conducted by RT-PCR Starter kit (Riobo Bio). PCR amplification was performed on ABI PRISM 7900 thermocycler using SYBR Premix Taq (Applied Biosystems, US). These primers for quantitative PCR were synthesized by Sangon Biotech (Shanghai) Co., Ltd. and summarized in Table S1. The relative transcriptional levels of each target mRNA was calculated normalized to human glyceraldehyde-3-phosphate dehydrogenase (GAPDH), which was analyzed by 2^−ΔΔCT^ method.

### Lactate detection and treatment

The lactate content in cell culture supernatant or tissues was measured by l-lactatic acid colorimetric assay kit (Cat No: E-BC-K044-M, ELAB science BioScientific Ltd. Elabscience) following the manufacturer’s instructions. For the exogenous lactate treatment, l-lactate (10 mM, Sigma-Aldrich, cat No. L6402) was performed based on the protocols.

### Proliferative ability and apoptosis analysis

The proliferative ability of cervical cancer cells in the co-culture was detected using by the CCK-8 assay kit (Dojindo Japan). In brief, cervical cells were seeded in 96-well culture plates. The optical density (OD) value was detected at 450 nm using an enzyme-mark reader (Multiskan FC, Thermo Fisher Scientific, Waltham, MA, USA). For the apoptosis of co-cultured cervical cancer, the apoptosis was analyzed by flow cytometry. Double staining by fluorescein isothiocyanate (FITC)-annexin V and propidium iodide (PI) was conducted using the FITC Annexin V Apoptosis Detection Kit (BD Biosciences) according to the manufacturer’s instruction.

### Enzyme‑linked immunosorbent assay (ELISA)

To determine the level of cytokines, the Granzyme B, IL-10, IL-2, TGF-β, and IFN-γ levels in the supernatant of CD8^+^ T cells were detected by ELISA Kit (Invitrogen).

### Cytotoxicity analysis

The T-cell mediated cytotoxicity towards cervical cancer cells was tested by LDH assay. After co-culture of cancer cells and CD8^+^ T cells for 7 h, the supernatants were collected to detect the lactate dehydrogenase (LDH) release amount. The supernatant was tested using the Cytotoxicity Detection Kit PLUS (cat no. 04744926001, Sigma-Aldrich) according to the manufacturer’s protocol.

### Surface PD-L1 analysis

Surface MCT1 level was conducted occupying the Flow cytometric assay. Cervical cancer cells were re-suspended in PBS buffer and stained by primary antibody APC anti-human MCT1 (Abcam, ab213524) according to standard protocols. To measure cell surface MCT1 expression, cytometry staining was performed according to standard protocols and analyzed on Cytoflex Flow Cytometer (Beckman Coulter Life Sciences). Data were analyzed using the FlowJo software.

### RNA‑binding protein immunoprecipitation (RIP)

RIP assay was conducted using the Protein A/G agarose beads conjugated by specific antibody or control IgG at 4 °C for 2 h. After 48 h post-transfection, cells were harvested and lysed by NP-40 lysis buffer (Beyotime, China). Protein-A/G agarose beads were binding with YTHDF1antibody for 3 h. The immunoprecipitation was performed by EZ-Magna RIP Kit (Millipore) according to the manufacturer’s protocol. After centrifugation, the supernatant was incubated. The extracted RNA from the immunoprecipitation and the precipitated RNA was determined by qRT-PCR analysis.

### Pull down

Biotin-labeled YTHDF1 probe and control probe were synthesized by GenePharma (Shanghai, China). Cervical cancer cells were lysed with lysis buffer and incubated by indicated probes. The cellular lysate was incubated with pre-coated magnetic beads to pull down the biotin-labeled RNA element. Lastly, the beads were washed with buffer and the complexes were purified by TRIzol (Takara, Dalian, China). Finally, the abundance of MCT1 blot was detected.

### RNA fluorescence in situ hybridization

To identified the subcellular location of YTHDF1 and MCT1 in cervical cells, the RNA-Fluorescence in situ hybridization (RNA-FISH) assay was performed. Two Cy3-labeled MCT1 probes and FAM-labeled YTHDF1 were synthesized by GenePharma (Shanghai, China). Hybridization was performed overnight with probes according to the manufacturer’s instructions. The images were captured on confocal laser scanning microscope (Olympus FV1000). 4,6-diamidino-2-phenylindole (DAPI) was used to stain the cell nucleus.

### RNA decay analysis

Cervical cancer cells were seeded into six-well plates with confluence of 90%. After transfection, the control (sh-NC or vector) and YTHDF1 knockdown (sh-YTHDF1) or overexpression (YTHDF1) cells were treated with 8 μg/ml actinomycin D (Act D) for 0, 3, 6 h. RNA was extracted from cells for qRT-PCR. The relative quantification level was identified by 2^−ΔΔCt^ method and normalized to GAPDH. The half-time time of MCT1 mRNA was calculated.

### Animal in vivo assay

Approximately 5 × 10^6^ mouse cervical cancer cells (U14) suspended in 100 μl (sh-NC, sh-YTHDF1-1) per mouse were injected into the flank of male C57BL/c mice. The assay had been approved by the Animal Ethics Committee of The Second Xiangya Hospital. The growth was measured every 4 days by monitoring the volumes, which was calculated by the formula: 0.5 × length × width × width. The mice were sacrificed after 4 weeks days and tumors were removed for further analysis.

### Statistical analysis

Statistical analysis was performed by using GraphPad Prism v9.0 (GraphPad, San Diego Statistical Analysis). The experiment was performed three times for average value. Statistical data was expressed as means ± standard deviation (SD). Comparison within different groups were calculated by Student’s *t*-test or variance (ANOVA) analysis on GraphPad Prism. Values of *p* < 0.05 were considered statistical significance.

## Results

### Elevated YTHDF1 indicated the poor prognosis and tumor infiltrating lymphocyte

**Fig. 1:**
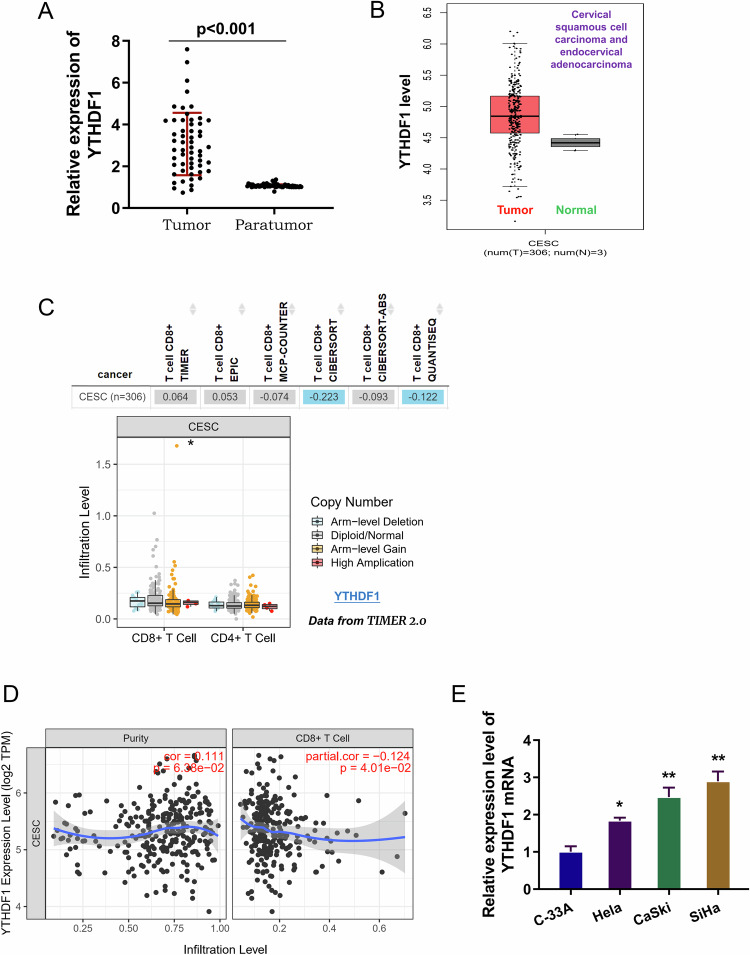
Elevated YTHDF1 indicated the poor prognosis and tumor infiltrating lymphocyte. **A** The level of YTHDF1 was detected in the cervical cancer samples. **B** In the public dataset (http://gepia.cancer-pku.cn/index.html), the YTHDF1 level increased the cervical cancer individuals. **C** In the TIMER database (http://timer.cistrome.org/), the YTHDF1 copy number altered the infiltration levels of CD8^+^ T cells. **D** TIMER were used to predict the correlation of YTHDF1 with CD8^+^ T cells in cervical cancer. **E** RT-PCR was performed to illustrate the YTHDF1 mRNA in the cervical cancer cells. *p < 0.05; *p* < 0.01.

In the cervical cancer samples, the level of YTHDF1 significantly elevated as comparing to the normal controls (Fig. 1A). In the public dataset (http://gepia.cancer-pku.cn/index.html), the YTHDF1 level also substantially increased the cervical cancer individuals (Fig. 1B). Tumor infiltrating lymphocyte (TILs) therapy has achieved good results in the treatment of solid tumors. To investigate the effect of YTHDF1 on the immune microenvironment, our research explored the link between immune cells and YTHDF1. In the TIMER database (http://timer.cistrome.org/), the YTHDF1 copy number altered the infiltration levels of CD8^+^ T cells (Fig. 1C). Data also showed that upregulated YTHDF1 mRNA in cervical cancer patients was strikingly negatively correlated to the levels of CD8^+^ T cells (Fig. 1D). In the cervical cancer cells, the YTHDF1 mRNA significantly upregulated (Fig. 1E). Overall, the data indicated that elevated expression of YTHDF1 was correlated with poor prognosis and tumor-infiltrating lymphocyte.

### YTHDF1 promoted the glycolysis, lactate accumulation and surface PD-L1 expression

**Fig. 2:**
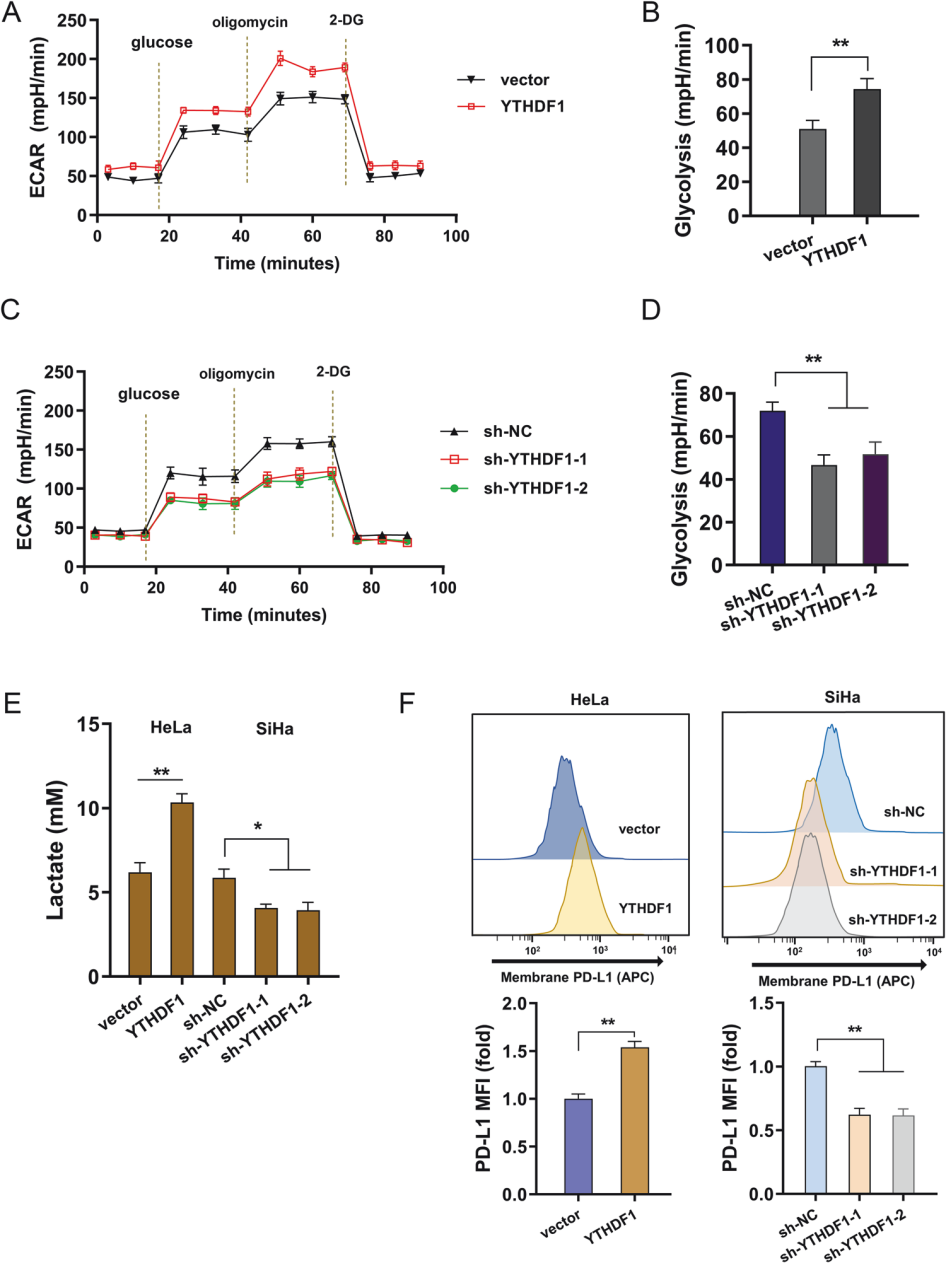
YTHDF1 promoted the glycolysis, lactate accumulation and surface PD-L1 expression. **A**–**D** Extracellular acidification rate (ECAR) was performed to detected the extracellular acidification rate of glycolysis. **E** The supernatant lactate expression was detected by l-lactatic acid colorimetric assay kit in cervical cancer cells with YTHDF1 silencing and control group. **F** The cellular surface PD-L1 expression on cervical cancer cells were detected using flow cytometry. *p < 0.05; **p < 0.01.

To explore the function of YTHDF1 on cervical cancer cells’ phenotypic characteristic, the following assays were performed. Firstly, the glycolysis analysis was performed by extracellular acidification rate (ECAR) assay. The extracellular acidification rate after glucose treatment indicates the glycolysis rate. The extracellular acidification rate showed that YTHDF1 overexpression promoted the glycolysis of cervical cancer cells, and YTHDF1 silencing inhibited the glycolysis (Figs. 2A–D). Moreover, the lactate in the culture medium was analyzed and results illustrated that YTHDF1 overexpression promoted the lactate enrichment, and YTHDF1 silencing reduced the lactate (Fig. 2E). Surface PD-L1 analysis revealed that YTHDF1 overexpression promoted the surface PD-L1 level, and YTHDF1 silencing decreased the surface PD-L1 level (Fig. 2F). Collectively, these data illustrated that YTHDF1 promoted the glycolysis, lactate accumulation and surface PD-L1 expression.

### YTHDF1 hampered the cytotoxic CD8^+^ T cell’s killing effect to cervical cancer cells

**Fig. 3:**
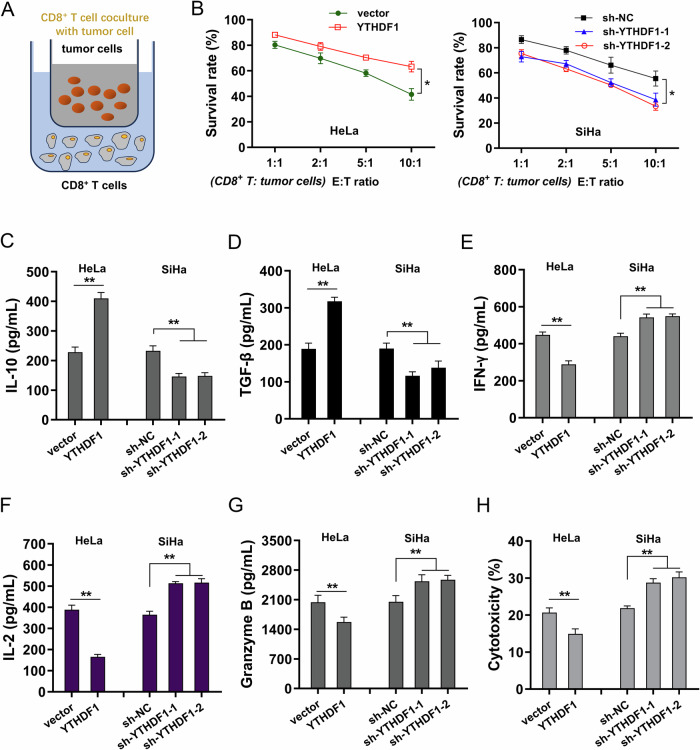
YTHDF1 hampered the cytotoxic CD8^+^ T cell’s killing effect to cervical cancer cells. **A** The coculture within activated CD8^+^ T cells and cervical cancer cells was constructed. **B** Survival analysis was performed by CCK-8 assay illustrated the survival rate of cervical cancer cells (HeLa, SiHa) in the coculture system in different *E*:*T* ratio (effector:target ratio) ranging from 1:1 to 10:1. **C**–**F** In the coculture within activated CD8^+^ T cells and cervical cancer cells (HeLa, SiHa), the quantitative analysis of immunosuppressive cytokines (IL-10, TGF-β) and immune effector cytokines (IFN-γ, IL-2) from activated CD8^+^ T cells was identified by ELISA kit. **G** The production of cytolytic granzyme B from activated CD8^+^ T cells was tested by ELISA kit. **H** The CD8^+^ T cells’ cytotoxicity assay based on LDH release was performed to determine the release of LDH into the coculture supernatants. *p < 0.05; *p* < 0.01.

To investigate the function of YTHDF1 on cytotoxic CD8^+^ T cell’s killing effect to cervical cancer cells, the coculture within activated CD8^+^ T cells and cervical cancer cells was constructed (Fig. 3A). Survival analysis illustrated that YTHDF1 overexpression promoted the survival rate of cervical cancer cells in the coculture system in different *E*:*T* ratio (effector:target ratio) (Fig. 3B). Subsequently, to test whether YTHDF1 regulated the immunosuppressive tumor microenvironment, the immunosuppressive cytokines (IL-10, TGF-β) and immune effector cytokines (IFN-γ, IL-2) were tested. Results indicated that YTHDF1 promoted the secretion of immunosuppressive cytokines (IL-10, TGF-β) (Figs. 3C, D), and reduced the secretion of immune effector cytokines (IFN-γ, IL-2) (Figs. 3E, F) among activated CD8^+^ T cells. Moreover, the production of cytolytic granzyme B was tested and results indicated that CD8^+^ T cells incubated with YTHDF1 high-expression transfected cervical cancer cells secreted significantly lower amounts of granzyme-B than that YTHDF1 silencing transfection (Fig. 3G). The T cells’ cytotoxicity assay based on LDH release indicated that the cytotoxic activity of CD8^+^ T cells against YTHDF1 high-expression transfected cervical cancer cells was markedly lower than that of cells transfected with controls (Fig. 3H). Correspondingly, the knockdown of YTHDF1 exerted the opposite effect. Taken together, YTHDF1 hampered the cytotoxic CD8^+^ T cell’s killing effect to cervical cancer cells.

### MCT1 was targeted by YTHDF1 in cervical cancer microenvironment

**Fig. 4:**
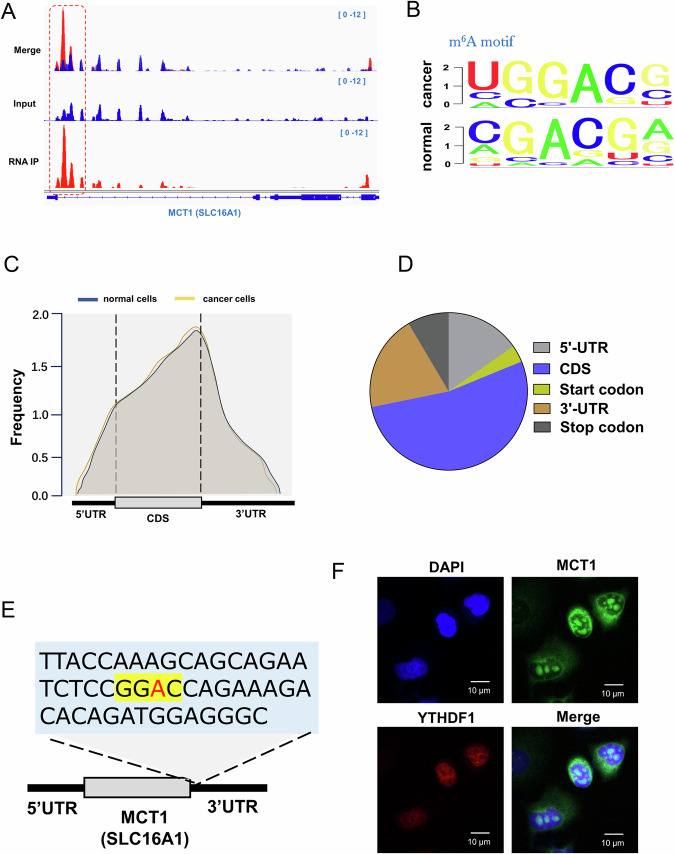
MCT1 was targeted by YTHDF1 in cervical cancer microenvironment. **A** The m^6^A modification on MCT1 (SLC16A1 gene) was shown by IGV (Integrative Genomics Viewer). **B** The m^6^A methylation motifs of cervical cancer and normal samples were shown. **C** Distribution of new m^6^A peaks in mRNA detected derived from the NCBI GEO dataset (Series GSE242071). **D** The m^6^A methylation distribution was showed, including 5′-UTR, CDS, 3′-UTR and start/stop codon. **E** The precise location of m^6^A methylation site on MCT1 (SLC16A1 gene). **F** RNA fluorescence in situ hybridization (FISH) reflected the co-location of YTHDF1 and MCT1 in the cervical cancer cells.

Given the immunosuppressive action mediated by YTHDF1, the regulatory mechanism in depth was investigated in following analysis. The m^6^A modification on MCT1 (SLC16A1 gene) was shown by IGV (Fig. 4A). The m^6^A methylation motifs of cervical cancer and normal samples were shown as sequence image (Fig. 4B). Derived from the NCBI GEO dataset (Series GSE242071), the abnormal m^6^A methylation correlated to tumor microenvironment (TME) in cervical cancer was exhibited (Fig. 4C). The m^6^A methylation distribution was also demonstrated, including 5′-UTR, CDS, 3′-UTR and start/stop codon (Fig. 4D). The precise location of m^6^A methylation site on MCT1 gene was accurately determined using diversiform assays (Fig. 4E). The co-location of YTHDF1 and MCT1 was investigated using RNA fluorescence in situ hybridization (FISH) and data indicated that YTHDF1 and MCT1 consistently distributed in the cytoplasm of cervical cancer cells (Fig. 4F). Taken together, the data illustrated that MCT1 was targeted by YTHDF1 in cervical cancer immune microenvironment.

### YTHDF1 potentiated MCT1 expression by enhancing MCT1 mRNA stability

**Fig. 5:**
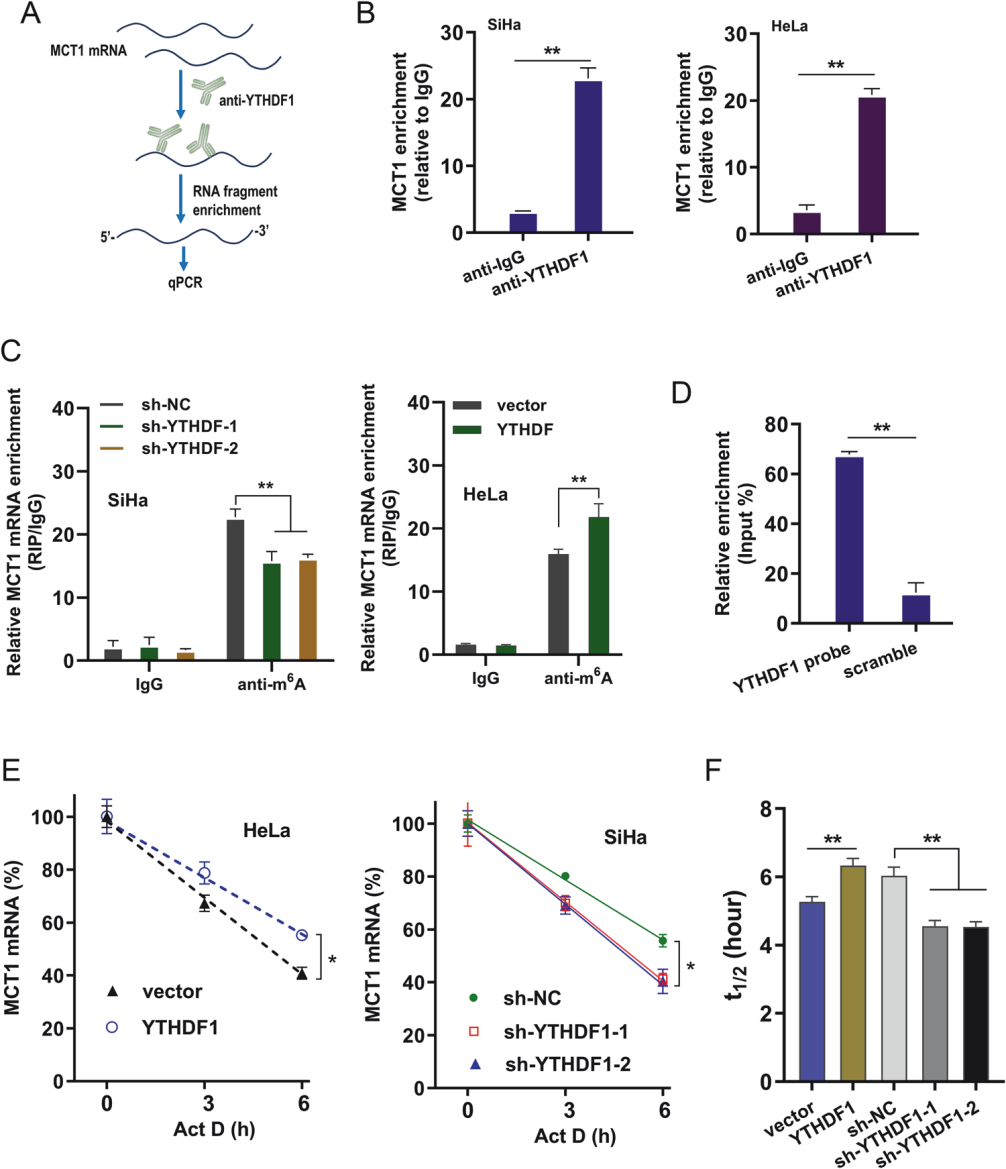
YTHDF1 potentiated MCT1 expression by enhancing MCT1 mRNA stability. **A** RIP-PCR assay was performed to test the binding within anti-YTHDF1 anti-body and MCT1 mRNA in cervical cancer. **B** The precipitated MCT1 mRNA enrichment was quantificationally analyzed by PCR. **C** RIP-PCR assay was performed in cervical cells (SiHa, HeLa) transfected with YTHDF1 overexpression (YTHDF1 or vector) or YTHDF1 knockdown (sh-YTHDF1 or sh-NC). **D** RNA pull-down assay was performed to confirm the binding of YTHDF1 and MCT1. **E**, **F** RNA stability analysis was performed upon Act D treatment. *p < 0.05; *p* < 0.01.

The immunotherapy targeting MCT1 is the most mainstream clinical tumor treatment technology. To verify how YTHDF1 regulated the fate of MCT1 mRNA, more assays were performed to identify it. RIP-PCR assay indicated that YTHDF1 strikingly bound with the MCT1 mRNA in cervical cancer (Fig. 5A, B). Moreover, to validate the regulation potential of YTHDF1 on m^6^A-binding to MCT1 mRNA, RIP-PCR assay illustrated that YTHDF1 overexpression promoted the binding of m^6^A to MCT1 mRNA, while YTHDF1 knockdown reduced the binding activity (Fig. 5C). RNA pull-down assay was performed to confirm the binding of YTHDF1 and MCT1 (Fig. 5D). RNA stability analysis revealed that YTHDF1 overexpression enhanced the MCT1 mRNA stability, while YTHDF1 knockdown reduced the MCT1 mRNA stability (Fig. 5E, F). In summary, the findings illuminated that YTHDF1 potentiated MCT1 expression by enhancing MCT1 mRNA stability.

### YTHDF1/MCT1 boosted the lactate accumulation and potentiated cervical cancer cells immune escape

**Fig. 6:**
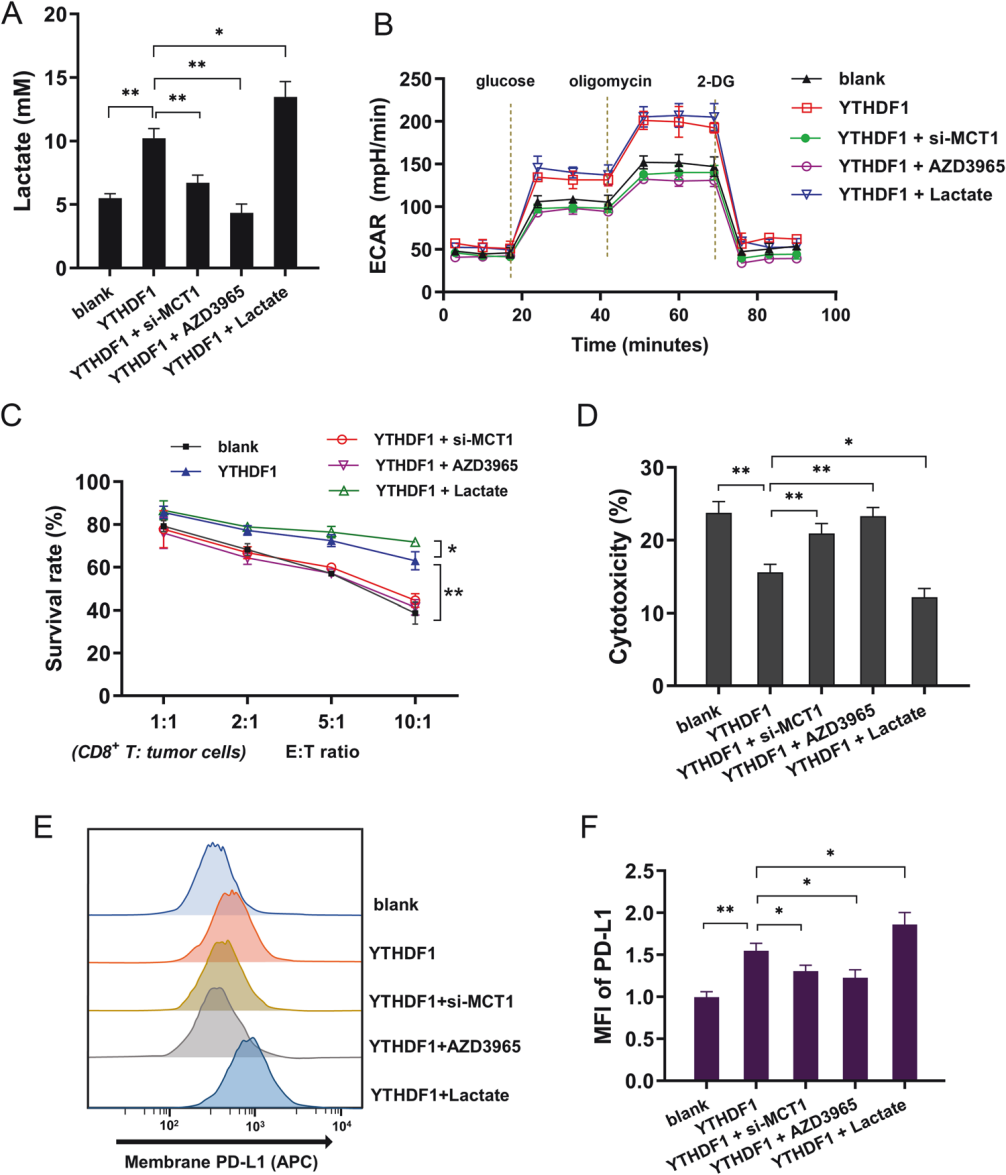
YTHDF1/MCT1 boosted the lactate accumulation and potentiated cervical cancer cells immune escape. **A** Lactate was detected by l-lactatic acid colorimetric assay kit in cervical cancer (SiHa) cells transfected with YTHDF1 overexpression plasmids (YTHDF1), MCT1 silencing (si-MCT1), MCT1 specific inhibitor (AZD3965) and exogenous lactate (Lactate, 10 mM l-lactate). **B** The ECAR was detected in the cervical cancer (SiHa) cells. **C** Survival analysis by CCK-8 exhibited the survival rate of cervical cancer cells (SiHa) in the coculture system in different *E*:*T* ratio (effector:target ratio) ranging from 1:1 to 10:1. **D** The CD8^+^ T cells’ cytotoxicity assay based on LDH release was performed to determine the release of LDH into the coculture supernatants. **E**, **F** The cellular surface PD-L1 expression on cervical cancer cells were detected using flow cytometry. *p < 0.05; *p* < 0.01.

The acidic environment formed by high concentration of lactate is crucial for tumor cell angiogenesis, metastasis and treatment resistance. Previous data indicated that YTHDF1 targeted MCT1 to regulate the MCT1 mRNA stability. Subsequently, rescue assays were performed to verify the regulative axis. Lactate analysis, glycolysis, survival analysis and surface PD-L1 analysis revealed that YTHDF1 overexpression promoted the lactate, glycolysis, survival analysis, and PD-L1 level. Besides, MCT1 knockdown (si-MCT1) and MCT1 specific inhibitor (AZD3965) repressed the lactate, glycolysis, survival analysis and PD-L1 level. Moreover, the exogenous lactate accelerated the lactate, glycolysis, survival analysis, and PD-L1 level (Fig. 6A–C, E, F). CD8^+^ T cells’ cytotoxicity analysis revealed that YTHDF1 overexpression repressed the survival rate of cervical cancer cells, and reduced the CD8^+^ T cells’ cytotoxicity. Besides, MCT1 knockdown (si-MCT1) and MCT1 specific inhibitor (AZD3965) promoted the leukomonocyte’s cytotoxicity (Fig. 6D). Moreover, the exogenous lactate inhibited the cytotoxicity. Therefore, these data suggested that YTHDF1/MCT1 boosted the lactate accumulation and potentiated cervical cancer cells immune escape.

### YTHDF1 silencing repressed the cervical cancer tumor growth and lactate in vivo

**Fig. 7:**
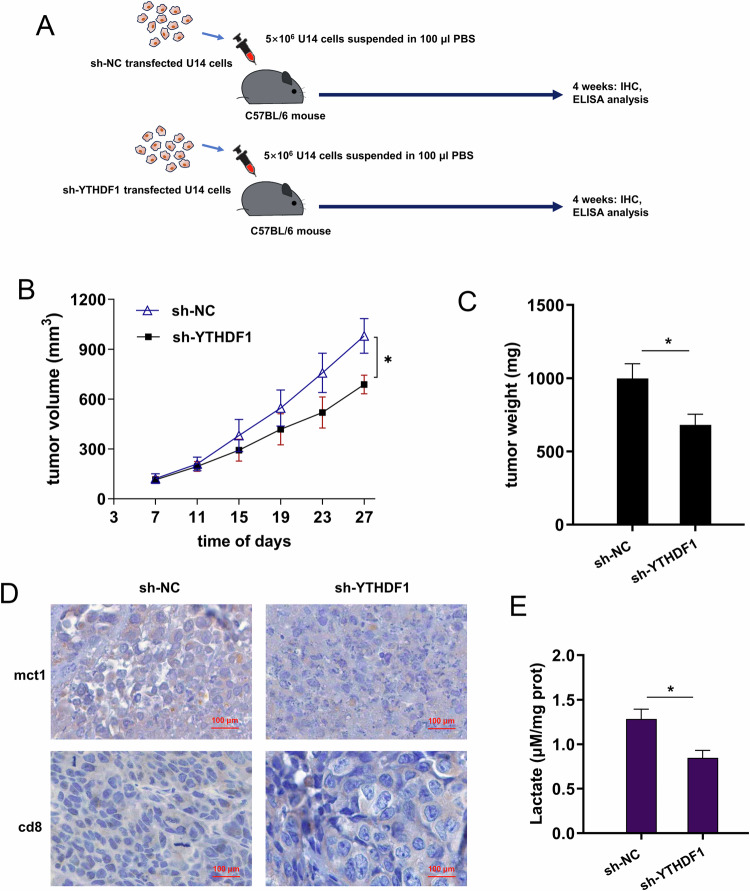
YTHDF1 silencing repressed the tumor growth and lactate in vivo. **A** The in vivo mice assay was performed using mouse cervical cancer cells (U14 cells with sh-NC, sh-YTHDF1). **B** Tumor volume and **C** tumor weight of cervical cancer were detected and calculated. **D** Immumohistochemical (IHC) staining revealed the positive mct1 and cd8 protein in the YTHDF1 silencing group and control group. **E** The in vivo lactate expression was detected by l-lactatic acid colorimetric assay kit in YTHDF1 silencing and control group. *p < 0.05.

To verify the role of YTHDF1 on cervical cancer tumor growth and lactate, the in vivo mice assay was performed using mouse cervical cancer cells (U14 cells with sh-NC, sh-YTHDF1) (Fig. 7A). In the volume calculation, the YTHDF1 silencing repressed the tumor volume of cervical cancer cells (Fig. 7B), as well as the tumor weight (Fig. 7C). Besides, the positive mct1 protein levels were reduced in the YTHDF1 silencing group, and the cd8 positive level increased (Fig. 7D). Besides, the in vivo lactate expression was detected, and results illustrated that YTHDF1 silencing inhibited the lactate expression, relative to respective controls (Fig. 7E). Therefore, these data suggested that YTHDF1 silencing repressed the cervical cancer tumor growth and lactate in vivo.

## Discussion

In the immune microenvironment, lactate plays critical roles in promoting tumor immune escape, mainly through local environment acidification, restrain activity of immune cells and immune inhibitory cells. These changes triggered by lactate transform the tumor microenvironment into a microenvironment conducive to tumor survival, growth, and uncontrolled proliferation, ultimately leading to immune escape of tumor cells [18, 19]. The formation of tumor immunosuppressive microenvironment is one of the six biological characteristics that promote tumor growth and metastasis.

In the present research, the findings revealed that YTHDF1 was remarkably upregulated in the cervical cancer tissue sample and cells. Clinically, high expression of YTHDF1 predicted unfavorable clinical outcomes of cervical cancer, which was negatively correlated with CD8^+^ T cell infiltration. Previous literature indicated that YTHDF1 could mediate the m^6^A modification on TRIM68 to accelerate cellular viability, migration and invasion of prostate cancer [20]. In triple-negative breast cancer, YTHDF1 is highly expressed in breast cancer tissue and cells, and knockdown of YTHDF1 significantly inhibits stemness and promotes the chemosensitivity of cancer cells [21]. Thus, it could be drawn a conclusion that YTHDF1 plays multi-dimensional oncogenic functions on cervical cancer.

Given that CD8^+^ T cells are the major cytotoxic immune lymphocytes, the killing effect of CD8^+^ T cells on cancer cells is tremendous important to defeat the tumorigenesis [22]. In the co-culture of tumor cells with CD8^+^ T cells, YTHDF1 overexpression promoted the survival rate of cervical cancer cells and reduced the apoptosis of cervical cancer cells. Besides, YTHDF1 overexpression attenuated the cytotoxic CD8^+^ T cell’s killing effect and reduced cytokine secretion, including IFN-γ, Granzyme-B, and Perforin. Therefore, the immunosuppressive role of YTHDF1 in cervical cancer immune microenvironment is identified in this research and data. Presently, the immunotherapy targeting programmed death ligand-1 (PD-L1)/programmed death 1 (PD-1) functions as the most mainstream clinical tumor treatment technology [18, 23]. However, the limited response to PD-L1/PD-1 immunotherapy is a major hindrance of checkpoint immunotherapy in human cancer [22].

Lactate is mainly produced by glycolysis of tumor cells, until recently is regarded as a main fuel oxidation of cancer cells and signal transduction. Cancer cells metabolize to produce lactate and maintain a high-lactate environment, which inhibits the function of T cells (particularly effector T cells) that attack cancer cells. Lactate can not only directly inhibit the activity of T cells, but also indirectly suppress the immune response by promoting regulatory T cells (Treg) [24]. The abundance of MCT1 on cancer cell’s surface is crucial for the lactate transport [25, 26]. Lactate and MCTs, especially MCT1, are critical contributors to tumor immune escape. MCT1 is encoded by the solute carrier 16 (SLC16) family of gene (SLC16A1). Here, our results indicated that YTHDF1 targeted the m^6^A site on MCT1 mRNA to determine its fate. YTHDF1 upregulated MCT1 expression by enhancing MCT1 stability mediated by m^6^A modified manner. There were numerous m^6^A sites on the MCT1 genomic sequence, and the m^6^A modification on 3′-UTR acted as the effective targeting point. Here, YTHDF1 bound with the m^6^A modification of MCT1 3′-UTR. In cervical cancer, Sui et al. reported that lactate exposure reduced the immune effect of dendritic cells on cervical cancer cells, and MCT1 silencing enhances the effect induced by sodium lactate exposure [27]. Recent studies have found that knockout of MCT1 gene can prevent regulatory T cells from taking up lactate, thereby slowing down tumor growth, which is a potential breakthrough for tumor gene therapy [28].

**Fig. 8:**
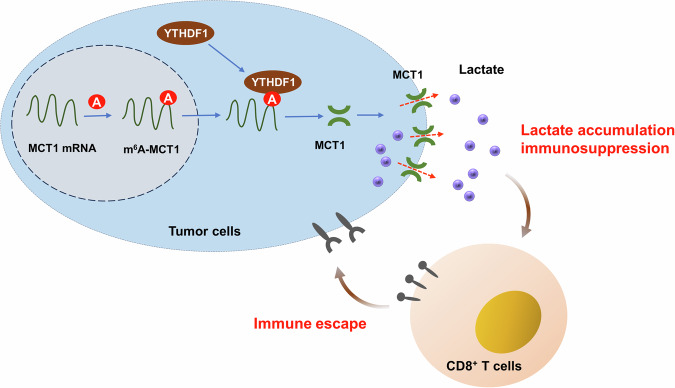
YTHDF1 deteriorates cervical cancer immune escape by inhibiting cytotoxic CD8^+^ T cells antitumor effect.

Collectively, our results revealed an oncogenic role played by YTHDF1 in cervical cancer through m^6^A/MCT1-dependent manner (Fig. 8). The m^6^A site on the MCT1 transcript targeting by YTHDF1 shows the significance of post-transcriptional control in tumor lactate accumulation. In conclusion, these findings unveil the immune escape-promoting effect of YTHDF1 in cervical cancer via lactate accumulation, which might illuminate a novel target for more precise immunotherapy.

## Supplementary information

blot

Table S1

## Data Availability

The datasets generated during and/or analyzed during the current study are available from the corresponding author on reasonable request.
