# Supplementary figures and images for: YTHDF1 boosts the lactate accumulation to potentiate cervical cancer cells immune escape
Source: Cell Death Dis. 2024 Nov 18;15(11):843. doi: 10.1038/s41419-024-07128-0 (PMC11573975; doi:10.1038/s41419-024-07128-0)

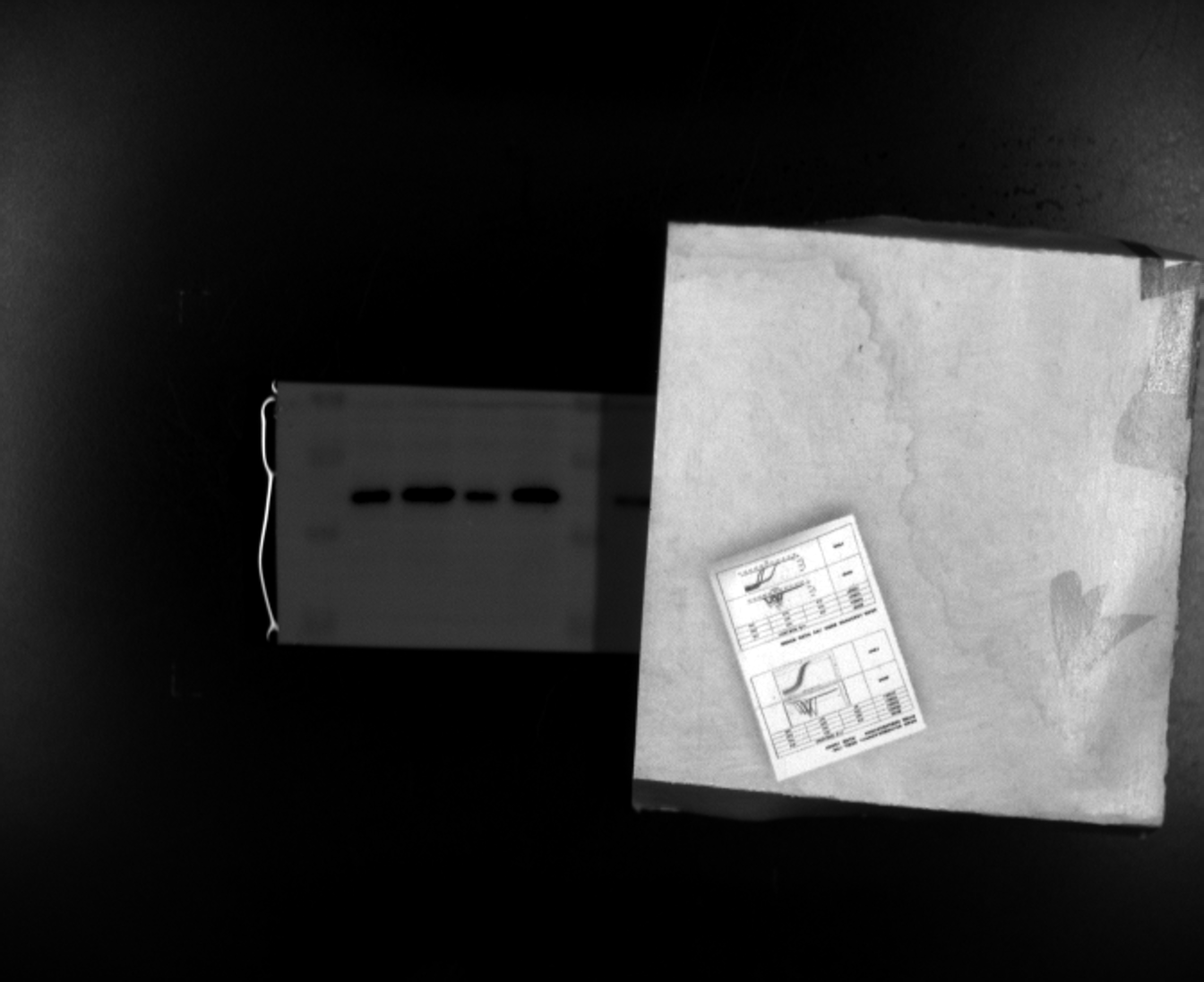

Supplement: Supplementary file 1 — blot [file 41419_2024_7128_MOESM1_ESM.tif]
