# Supplementary material for: YTHDF1 boosts the lactate accumulation to potentiate cervical cancer cells immune escape
Source: Cell Death Dis. 2024 Nov 18;15(11):843. doi: 10.1038/s41419-024-07128-0 (PMC11573975; doi:10.1038/s41419-024-07128-0)
Supplement: Supplementary file 2 — Table S1 [file 41419_2024_7128_MOESM2_ESM.docx]

**supplementary Table S1**. qRT-PCR primers sequences and shRNA sequences.

|  | Sequences |
| --- | --- |
| YTHDF1 | F, 5’- ATACCTCACCACCTACGGACA-3’  R, 5’-GTGCTGATAGATGTTGTTCCCC-3’ |
| MCT1 | F, 5’-GGTGGAGGTCCTATCAGCAGT-3’  R, 5’-CAGAAAGAAGCTGCAATCAAGC-3’ |
| sh-YTHDF1-1 | 5’-GTTCGTTACATCAGAAGGATA-3’ |
| sh-YTHDF1-2 | 5’- CGGTGGGACAAATGTGAACAT-3’ |
| GAPDH | F, 5’-CAGGAGGCATTGCTGATGAT-3’  R, 5’-GAAGGCTGGGGCTCATTT-3’ |
| b-actin | F, 5’- CATGTACGTTGCTATCCAGGC -3’  R, 5’- CTCCTTAATGTCACGCACGAT-3’ |
